# Experimental evidence for a suction–biting performance tradeoff in Mesoamerican cichlids

**DOI:** 10.1242/jeb.252347

**Published:** 2026-09-09

**Authors:** Khalil T. Russell, Peter C. Wainwright

**Affiliations:** Department of Evolution & Ecology, University of California, Davis, Davis, CA 95616, USA

**Keywords:** Cichlidae, Ecology, Tradeoff, Feeding, Behavior, Performance, Functional morphology

## Abstract

Tradeoffs can occur when multiple functions are shared by a single mechanical system, where changes to the system that improve the performance of one function necessarily lead to a decrease in performance of a second function. The teleost oral jaws are a complex mechanical system, and a multifunction tradeoff is thought to exist between the opposing functions of suction-based and biting-based feeding. We experimentally measured suction- and biting-based feeding performance across 10 heroine cichlid species spanning a range of ecomorphological diversity to test for the presence of a suction–biting tradeoff. We found an inverse relationship between suction-based and biting-based feeding performance across species, as well as significant relationships between performance and morphological features of the oral jaws, where larger oral-jaw gape and greater protrusion were associated with improved suction-feeding and decreased benthic-feeding performance. We observed a fivefold range among species in biting rates during benthic-feeding trials, with a twofold range observed among benthic-feeding specialists, and propose that diversity in the properties of attached prey has generated diversity among biting-based specialists reliant on high-force bites versus those reliant on high biting rates.

## Teaser

**Summary:** Experimental assessment of multivariate suction and biting performance reveals a multifunction tradeoff linked to diet and morphology, with unexpected diversity among biting specialists.

## INTRODUCTION

Tradeoffs can occur when a single mechanical system performs multiple functions, where changes to the system that improve one aspect of performance decrease performance on a second axis (Anderson et al., 2014; Corbin et al., 2015; Garland et al., 2022). This phenomenon is thought to have profound effects on the trajectory of trait evolution, limiting the viability of certain trait combinations and biasing patterns of ecological, morphological and functional diversification (Burress and Muñoz, 2023; Corn et al., 2021; Sansalone et al., 2024; Sheftel et al., 2018). The teleost oral jaws are a complex, multifunctional system that fishes use to perform diverse ecological tasks (e.g. egg cleaning, nest building, fighting, substrate sifting, respiration), and whose diversification has enabled teleosts to occupy a wide range of trophic niches (Konow et al., 2008; Olvera-Ríos et al., 2023; Říčan et al., 2016). Among the most studied functions of the teleost oral jaws is suction-based feeding, which involves a coordinated action of the oral jaws as part of a larger feeding apparatus used to capture evasive, free-moving prey by rapidly expanding the mouth and buccal cavity to generate a flow of water that carries the prey into the mouth (Alfaro et al., 2001; Ferry et al., 2015; Lauder, 1985). However, not all prey is free moving or effectively captured with suction, and many fishes have evolved the ability to feed on plants and animals that are anchored to the substrate using biting actions in which the jaws are used to grip and pull, or scrape prey from their holdfast. A mechanical tradeoff in the jaws is thought to exist between suction-based and biting-based feeding modes in teleosts (Ferry-Graham et al., 2002; Wainwright and Richard, 1995; Westneat, 1994).

**Fig. 1.**
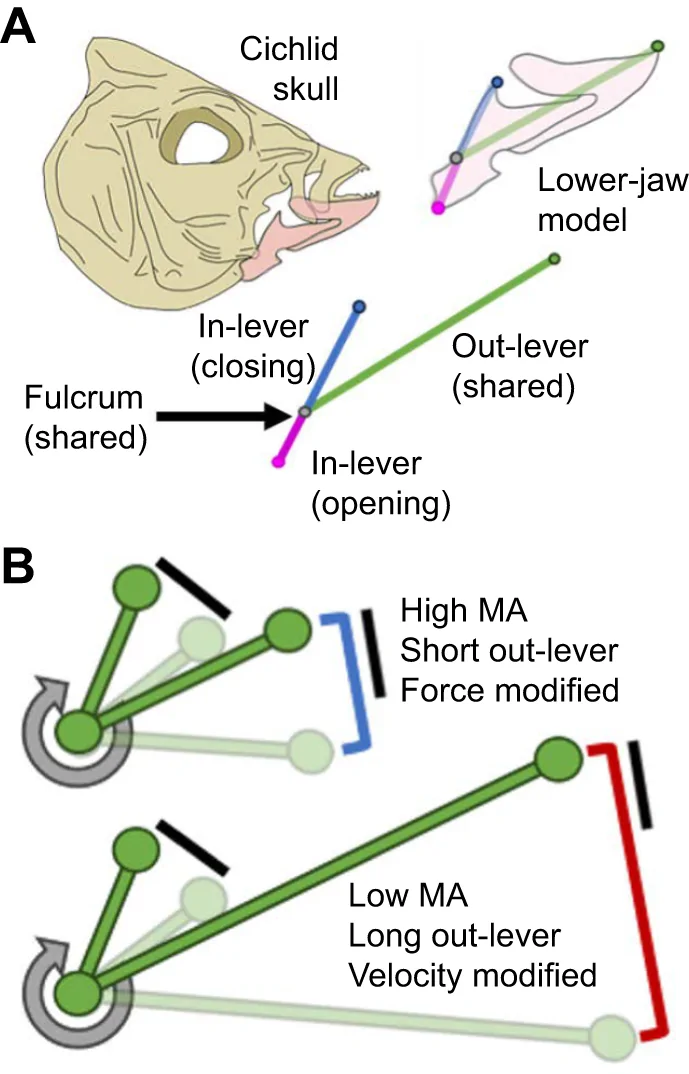
**The teleost jaw.** (A) Lever model of the teleost lower jaw. Left: the cichlid skull. Right: lower jaw lever model. Bottom: depiction of the relationship between the closing in-lever, the opening in-lever (pink), the shared out-lever (green) and the fulcrum at the articular–quadrate joint. (B) High and low mechanical advantage (MA) levers depicting the relationship between input motion (black line) and output motion (blue and red lines, respectively). Low MA levers enhance motion transfer compared with high MA levers, which are better at transferring forces.

The teleost oral jaws can be modeled as a set of interconnected levers with a prominent role played by the mandible, modeled as a single out-lever connected to two in-levers by a shared fulcrum (Westneat, 2003; Fig. 1A). The two in-levers reflect the functions of jaw opening and closing and involve the actions of distinct muscular systems. Levers with a shorter out-lever relative to the in-lever have high mechanical advantage, which results in greater force transmission. In contrast, levers with a longer out-lever have lower mechanical advantage and propagate more motion through the jaws. Low mechanical advantage in the mandible is expected to enhance jaw mobility, resulting in faster rates of mouth opening and closing for given input motions (Fig. 1B). Mechanical advantage may also influence jaw excursion and muscle operating lengths, potentially creating additional tradeoffs between muscular force production and range of motion (Polet and Labonte, 2024). Greater force transmission is expected to be advantageous for feeding modes that rely on biting, whereas greater mandibular mobility is advantageous for suction feeding, which involves rapid expansion of the buccal cavity to create negative pressure that draws water and prey into the mouth. Modeling efforts indicate that the mechanical tradeoff inherent to levers should drive a tradeoff between biting force and jaw mobility (De Schepper et al., 2008; Westneat, 1994). Ecomorphological studies motivated by these expectations have often shown that fishes reliant on biting-based feeding strategies have higher mechanical advantage in the jaw-closing lever ratio than taxa that primarily feed by suction (Arbour et al., 2020; Cooper et al., 2016; Turingan, 1994; Wainwright et al., 2004; Westneat, 1994, 1995).

Despite the theoretical and ecomorphological support, it has proven more challenging to link jaw mechanical advantage to feeding performance. Indeed, one study found no suction–biting tradeoff among three catfish species, which varied considerably in adductor muscle mass and hence bite force (Van Wassenbergh et al., 2007). This study emphasized that suction and biting performance both have a more complex basis than just mandible mechanical advantage, and, like other studies of fish feeding performance (Camp et al., 2015; Holzman et al., 2012; Olsen et al., 2017), suggests the importance of considering several morphological aspects of the feeding mechanism beyond the mandibular levers if one aims to understand the basis of feeding performance. Furthermore, whereas the suction–biting tradeoff is commonly thought to be a tradeoff between high-force biting and high-velocity suction-feeding strikes, the diverse mechanical characteristics of attached prey may promote commensurate diversity among attached-prey specialists. For example, whereas high bite forces may be important for biting species that crush hard prey with their oral jaws, such as mollusks, species that crop filamentous algae, scrape periphyton from hard surfaces or tear material from aquatic vegetation may not need as high biting forces. In the present study, we experimentally investigated the suction–biting tradeoff in a system where we expect feeding-mode reliance in nature to be related to the mechanical properties of the mandible, and where attached-prey specialization spans multiple types of attached prey.

The Cichlidae are a successful clade of teleosts renowned for their ecomorphological diversity. Cichlid diversification features repeated transitions between suction-based evasive prey capture and biting-based capture of attached prey (Burress and Muñoz, 2021; Hulsey and García De León, 2005; Říčan et al., 2016). Within this large family, heroine cichlids are a Mesoamerican radiation spanning a diverse range of trophic ecomorphologies (Olvera-Ríos et al., 2023). Previous work has documented considerable trophic versatility across the group, with evasive-prey specialists reliant primarily on suction-based feeding behaviors exhibiting the ability to feed on attached prey using biting-based feeding behaviors (Russell and Wainwright, 2026). In the present study, we measured feeding performance on prey requiring suction-based and biting-based feeding modes in 10 ecologically diverse species of heroine cichlids. We also measured oral jaw morphology, including the lever mechanics of the mandible, and related these traits to variation in feeding performance.

Although few studies have directly tested for it, and limited support has been found for a suction–biting performance trade-off (Bouton et al., 1998), this expectation remains a core tenet in fish feeding biomechanics (Deakin et al., 2022; Olivier et al., 2021; Oufiero et al., 2012). Thus, we expected an inverse relationship between suction-based and biting-based feeding performance. We also predicted that variation in feeding performance would be significantly correlated with variation in oral jaw morphology, with larger gapes and more protrusible jaws associated with suction-based feeding performance and larger adductor muscles with greater force transmission in the closing lever system of the mandible characterizing biting-based feeding performance. Finally, we also explored potential variation in feeding performance among attached-prey specialists and relate this variation to diversity in their preferred prey.

## MATERIALS AND METHODS

### Choice of study taxa

**Fig. 2.**
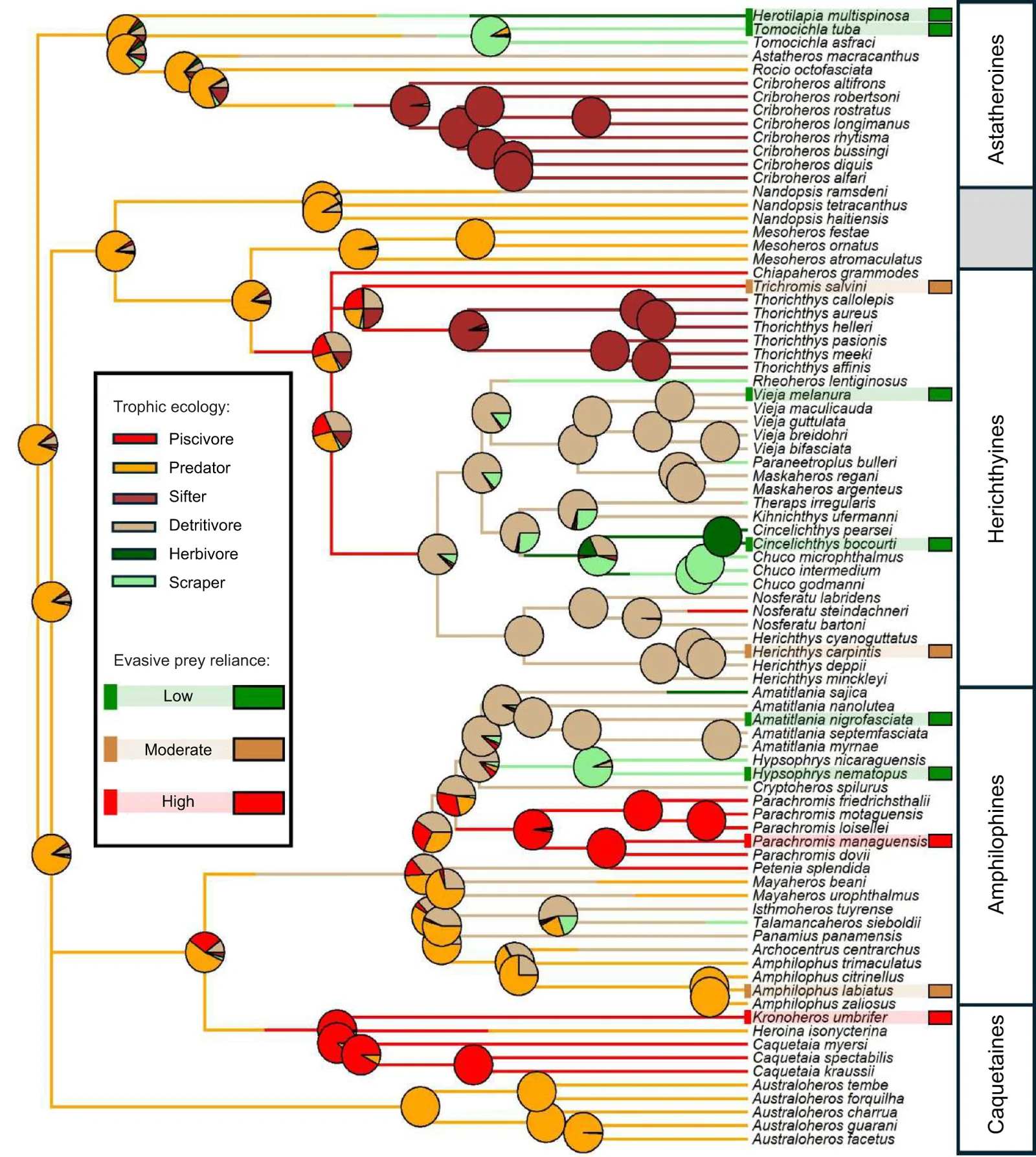
**Phylogeny of the Heroini overlaid with a randomly chosen simulation from an ancestral-state reconstruction of trophic ecology.** Pie charts at nodes summarize 100 simulations. Phylogeny is from McGee et al. (2020), trophic ecology from Říčan et al. (2016). Species experimentally assessed in the present study are in boxes colored by their ecological reliance on evasive prey: green, low; tan, moderate; red, high.

We reconstructed the history of trophic ecology among the Heroini using a comprehensive time-calibrated molecular phylogeny of Cichlidae (McGee et al., 2020), which was reconstructed from multiple sources of previously published DNA sequence data and provides estimates of relative divergence times (Fig. 2). We extracted the Heroini from the larger cichlid phylogeny and overlaid trophic states from Říčan et al. (2016). Trophic states were determined by Říčan et al. (2016) through a combination of volumetric gut content analyses following the methods used by Winemiller et al. (1995) and several field observation trips conducted between 2002 and 2014 (Říčan et al., 2016).

Ecomorphotypes were assigned for each species based on their predominant diet and food-gathering mode; this was obtained from the literature, most notably López-Fernández et al. (2013), who categorized neotropical cichlid ecomorphology based on morphological analysis, and the compendium of the biology of Middle American Cichlids (Stawikowski and Werner, 1998). The ecomorphs we use in our study are as follows: (1) piscivores – feed primarily on fishes; (2) predators – feed on a combination of small fishes and large invertebrates; (3) detritivores – primarily consume small invertebrates and fine detritus particles; (4) plant-eaters/herbivores – primarily consume living vegetation (higher plants and/or algae); (5) scrapers – feed on periphyton, composed of both small animals and vegetable material; (6) sifters – feed primarily by sifting small, buried invertebrates from mouthfuls of soft, small-grained sediment. We treated trophic state as a discrete character and estimated its evolution in the Heroini using the ‘ape’ package (v5.8.1; https://CRAN.R-project.org/package=ape; Paradis and Schliep, 2019) in R (*n=*100 simulations). We estimated ancestral states using maximum likelihood under an Equal Rates model of trait evolution using the ace() function from the ape package.

Based on this reconstruction we selected species for use in performance experiments from a range of trophic ecologies and phylogenetic subclades within the Heroini such that each ecomorph was represented by more than one evolutionary origin, if possible. Ecological reliance on evasive prey ranges from 3% or less (*Amatitlania nigrofasciata*, *Herotilapia multispinosa*, *Neetroplus nematopus*, *Tomocichla tuba*) to over 90% (*Parachromis managuensis*, *Kronoheros umbriferus*; Table 1). Based on our trophic state reconstruction and published analyses of the evolutionary history of diet in this clade (Fig. 2; Říčan et al., 2016), we included three independent transitions to specialized piscivory: caquetaines – *K. umbriferus*, amphilophines – *P. managuensis*, herichthyines – *Tomocichla salvini*. We also included two independent transitions to specialized algae/periphyton scraping (astatheroines – *T. tuba*, amphilophines – *N. nematopus*), and two to strict herbivory (astatheroines – *H. multispinosa*, hericthyines – *Cincelichthys bocourti*). Groups of 4–6 individuals of each species were sourced from commercial suppliers and used during feeding trials.

**Table 1.** Trophic ecology of the study species

Reliance on evasive prey – categorical	Species	Reliance on evasive prey – numerical (%)	Citation
Low	*Amatitlania nigrofasciata*	1	Gestring and Shafland (1997)
	Cincelichthys bocourti*	2	Soria-Barreto et al. (2019)
	*Herotilapia multispinosa*	1	Bussing (1993)
	*Neetroplus nematopus*	0	Winemiller et al. (1995)
	*Tomocichla tuba*	1	Winemiller et al. (1995)
	*Vieja melanurus*	3	Soria-Barreto et al. (2019)
Moderate	*Amphilophus labiatus*	50	Ariasari et al. (2018)
	*Herichthys carpintis*	25	Perez-Miranda et al. (2019)
	*Trichromis salvini*	48	Chavez-Lomeli et al. (1988)
High	*Kronoheros umbriferus*	90	Estimated from morphology
	*Parachromis managuensis*	92	Gestring and Shafland (1997)

Reliance on evasive prey information is primarily from Hulsey and García De León (2005). *Kronoheros umbriferus* ecology was estimated from trophic and morphological characterization by Říčan et al. (2016). *Cincelichthys bocourti ecology was estimated from gut content analysis of the sister species *Cincelichthys pearsei.*

**Fig. 3.**
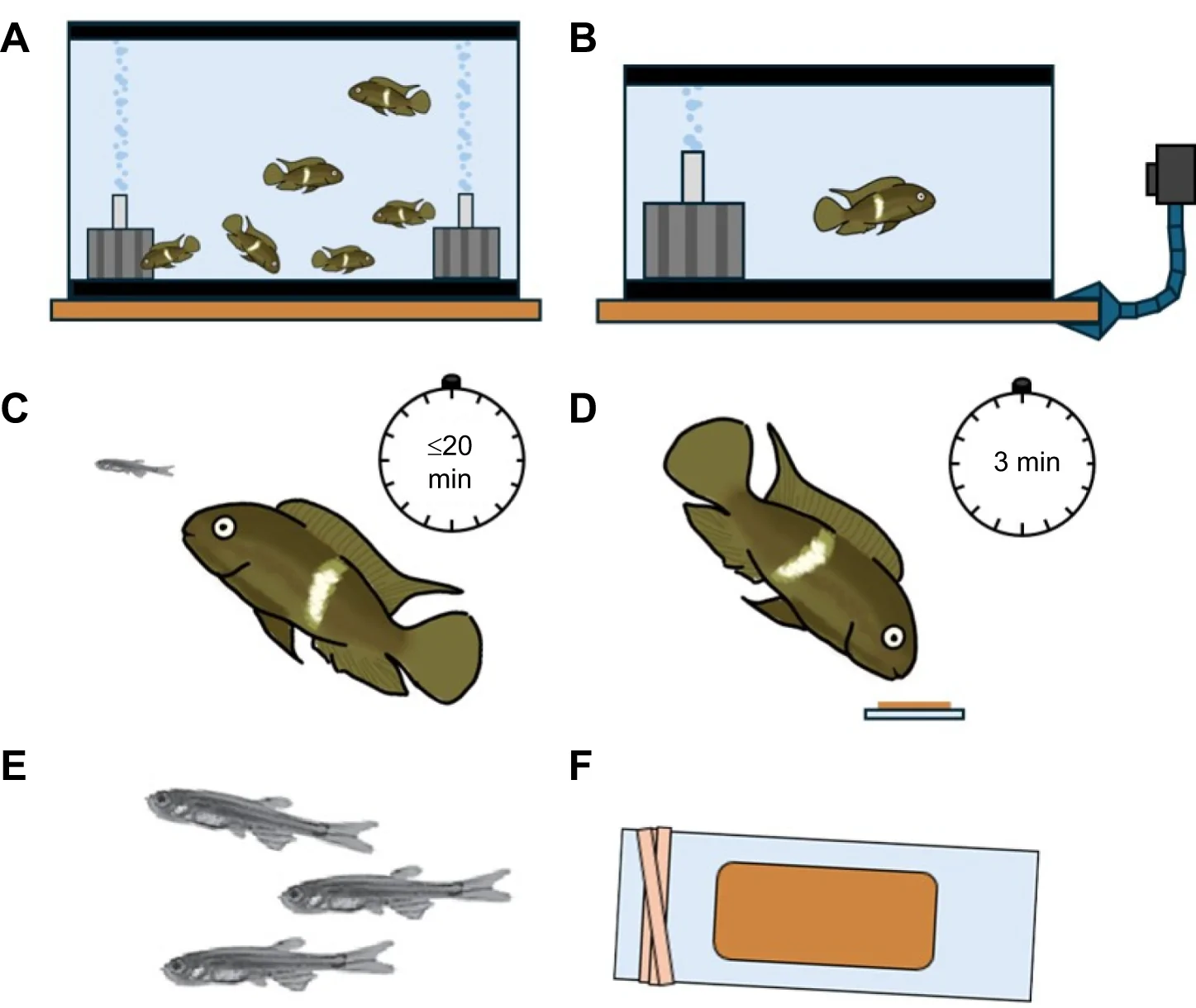
**Experimental set-up for measures of feeding performance in heroine cichlids.** (A) Prior to use in trials, subjects were housed in single-species 109 l aquaria. (B) during the experimental trial period, subjects were housed singly in 40 l aquaria. All trials were performed in these aquaria and via GoPro or a similar camera attached externally. (C) To measure suction-feeding performance, subjects were recorded capturing three evasive prey for no more than 20 min. (D) To measure benthic-feeding performance, subjects were recorded feeding on experimental attached food for 3 min. (E) Suction-feeding experimental treatment, consisting of three 2–4 week old zebrafish larvae added to experimental aquaria via dipnet. (F) Benthic-feeding experimental treatment, consisting of a gelatin/fish food mixture set on a microscope slide.

Subjects were group-housed in single-species pre-trial aquaria (109 l; Fig. 3A) at 24°C for approximately 4 weeks before participation in performance trials. During this period, subjects were fed primarily with Hikari Cichlid Staple pellet food. They were also exposed to the treatment foods used in the performance trials several times during this period.

This research was conducted under UC Davis IACUC protocol 23818.

### Feeding performance trials

Feeding performance trials were conducted in 40 l aquaria (Fig. 3B). Subjects were housed individually in aquaria that were visually isolated by opaque dividers. All performance trials were recorded with GoPro or similar video cameras (AKASO, Crosstour). Performance trials were conducted until five successful trials of each treatment were completed for each subject. For benthic-feeding trials, a successful trial was defined as any trial in which the subject took at least one bite of the attached prey. Successful suction-feeding trials we defined by capture of all evasive prey used during the trial. We excluded several attempted trials from analysis for various reasons.

In total, we present data on 59 individual subjects across 11 species (4–6 subjects per species). For both suction feeding and benthic feeding, we elected to characterize performance by measuring multiple outcomes of the trial, rather than a single overall measure of performance. We use a multivariate approach because we recognized multiple components of feeding performance in both trial types and felt this approach gave us the best chance of understanding specifically what distinguished the species.

Suction-feeding performance trials consisted of three zebrafish larvae (age: 2–4 weeks post-hatch; mean length: 12 mm) added together by dipnet to the trial aquarium. Subjects were given up to 20 min to capture all three larvae (Fig. 3C,E). After each successful trial, the video recording was reviewed and scored for the total number of strikes taken to capture all three zebrafish, the number of strikes taken until the first successful capture, the strikes taken between the first and second capture, and the strikes taken between the second and third capture. Additional measures were calculated for each subject from these variables, including: the perfect trial rate (i.e. the proportion of trials where the zebrafish prey were captured in only three strikes), the perfect strike rate (i.e. the proportion of successful captures requiring only a single strike) and the perfect strike rate for each prey captured (first, second and third).

Benthic-feeding performance trials were performed by exposing the subject to an experimental food designed to elicit a benthic-feeding response (Fig. 3D). The treatment consisted of a mixture of gelatin powder and crushed Hikari Cichlid Staple combined with boiling water and pipetted in ∼3×1 cm thin patches onto microscope slides (Fig. 3F). The slides were allowed to cool and set in a refrigerator for approximately 24 h. The dry mass of each food slide was recorded following the setting period, then the slides were rehydrated with water and Garlic Guard for 10 min. Garlic Guard is an additive meant to stimulate the fish's appetite.

Benthic-feeding slides were then placed into the trial aquaria for up to 10 min. The subjects were observed and were given 3 min to feed after the first bite was observed. Following the 3 min feeding period, the slide was removed and allowed to dry in the refrigerator for an additional 24 h before being weighed a second time. The number of bites taken was counted from each trial video. We also prepared and handled a set of control slides identically to the treatment slides. This was done to assess any mass change which may have occurred as a result of factors other than the subject's feeding (e.g. gelatin dissolving into the aquarium water).

The total mass consumed by the subject in each trial was calculated as the difference in dry mass of the slide before and after the trial, minus the mean mass change for the control slides for that round of trials. The total mass consumed was then divided by the body mass of the fish and this value was used in statistical analyses. The bite size was defined as the mass of food consumed by the fish per bite as a proportion of the subject's total body mass. We calculated bite size by dividing the mass consumed during each trial by the total number of bites taken for that trial, then dividing this value by the subject's body mass.

### Morphological measurements

Following the feeding trials, the subjects were euthanized with a 30 min exposure to a 7% solution of tricaine methane sulfonate. The following morphological characteristics were measured on fresh specimens: maximum gape height, maximum gape width, upper jaw protrusion distance and standard length (Table S1). Specimens were then fixed in 10% buffered formalin and stored in 70% ethanol. Body mass was measured from preserved specimens. Sections A1, A2 and A3 of the left adductor mandibulae muscles were dissected as a single mass from each specimen and weighed. Specimens were then cleared and stained according to Wassersug (1976) and Dingerkus and Uhler (1977). Mechanical advantage measurements were taken from the mandibles of cleared and stained specimens: closing in-lever length, opening in-lever length and out-lever length (Table 2).

**Table 2.** Loadings for the first and second principal components of the suction-feeding performance principal components analysis (PCA)

Variables	PC1 loadings	PC2 loadings
Total strikes	0.400	0.124
Strikes – prey 1	0.316	0.091
Strikes – prey 2	0.320	0.434
Strikes – prey 3	0.321	−0.402
Perfect trial rate	−0.333	0.109
Perfect capture – prey 1	−0.322	−0.181
Perfect capture – prey 2	−0.333	−0.297
Perfect capture – prey 3	−0.265	0.699
Perfect strike rate	−0.372	0.064

Principal component (PC)1=60.4%, PC2=12.4%.

### Statistical analysis

We analyzed gape size, defined as the mean of the vertical and horizontal oral jaw gape distances, as well as protrusion distance and adductor size. Gape radius, protrusion distance and lever arm lengths were analyzed as a proportion of standard length, and adductor mandibulae size as the ratio of the adductor mass to the specimen's total body mass. Closing mechanical advantage is the ratio between the closing in-lever and the out-lever, and opening mechanical advantage is the ratio between the opening in-lever and the out-lever.

We performed four principal components analyses (PCA) on scaled data. Continuous variables were standardized using the R function scale(), which subtracts the variable mean and divides by the standard deviation, resulting in variables with mean 0 and standard deviation 1. One PCA included only morphological data, one included all three benthic-feeding performance variables, one included all nine suction-feeding performance variables and one included all three benthic-feeding performance variables (bite count, bite size and mass consumption) along with the three suction performance variables that best summarized the full suite of nine variables: total strikes per trial, perfect trial rate and perfect single-capture rate. PCA were performed on the mean variable values of individual subjects, and species means were calculated by averaging principal component coordinates across individuals. We regressed individual variables and principal components axes using phylogenetic least squares (PGLS) regression.

## RESULTS

### Ancestral state reconstruction

We inferred several transitions between evasive (piscivore, predator) and attached (scraper, herbivore) prey, commensurate with findings from previous work (Říčan et al., 2016). Our results here provide context to convergent patterns in ecomorphology, behavior and performance across our sampled taxa. Notably, we show that each of the specialized piscivores, herbivores, scrapers and detritivores represent independent evolutionary transitions to their trophic state (Fig. 2).

### Suction-feeding performance

**Fig. 4.**
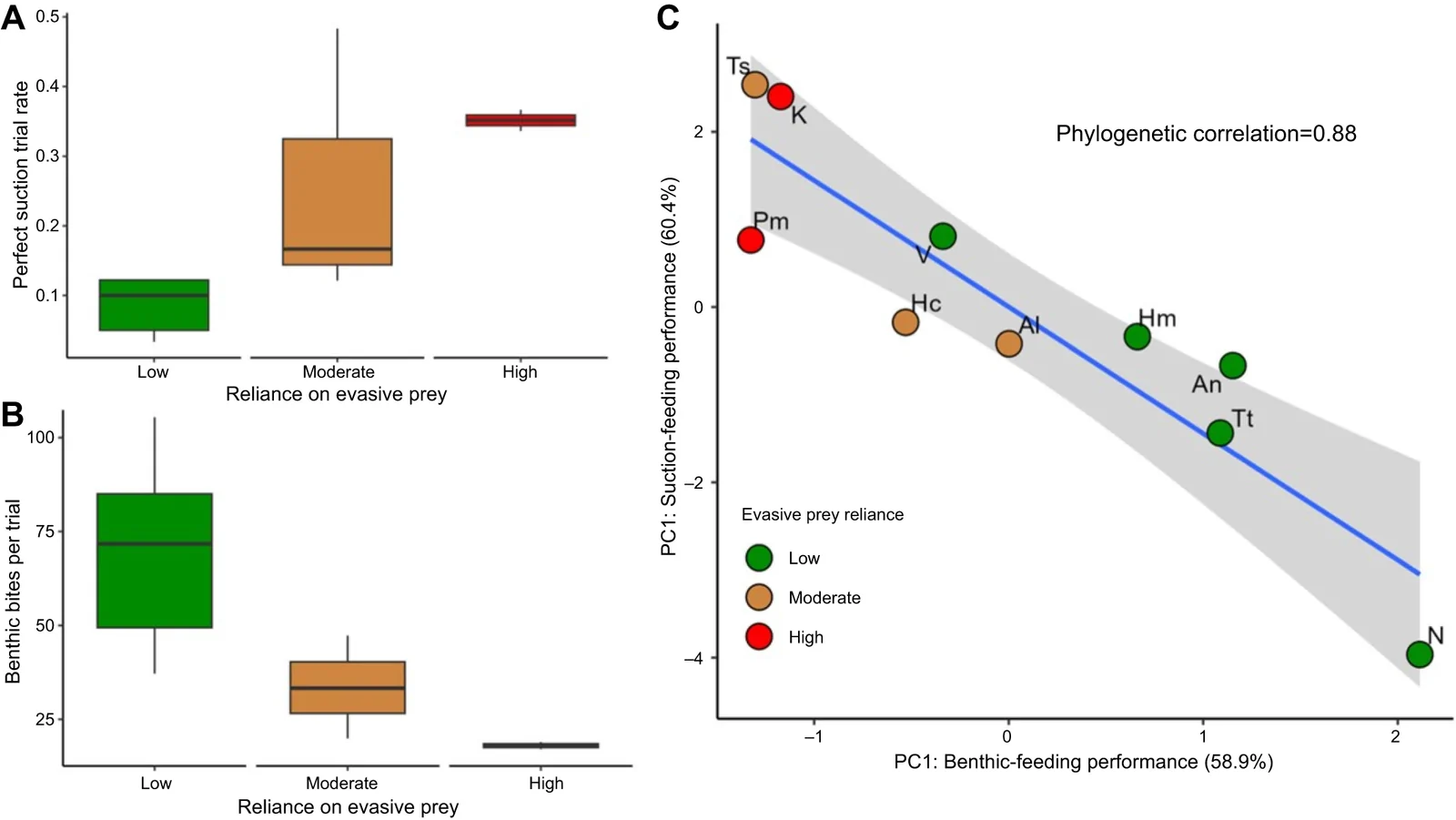
**Feeding performance in heroine cichlids.** (A) The rate of perfect suction trials, where three evasive prey are captured with one strike each, is positively correlated with ecological reliance on evasive prey. (B) The number of benthic bites per trial is negatively correlated with reliance on evasive prey. Box plots show median, upper and lower quartiles and 1.5× the interquartile range; colors are as in C. (C) Tradeoff between suction-feeding performance and benthic-feeding performance, with PC1 values from principal components analysis (PCA). Ts, *Trichromis salvini*; K, *Kronoheros umbriferus*; Pm, *Parachromis managuensis*; V, *Vieja melanurus*; Hc, *Herichthys carpintis*; Al, *Amphilophus labiatus*; Hm, *Herotilapia multispinosa*; An, *Amatitlania nigrofasciata*; Tt, *Tomocichla tuba*; N, *Neetroplus nematopus*. Note, we were unable to quantify suction-feeding performance in *Cincelichthys bocourti*.

The total number of strikes needed to capture three zebrafish larvae varied by species from a low of 3.9 (*K. umbriferus* – specialized piscivore) to a high of 10.8 (*N. nematopus* – specialized scraper). However, the mean number of strikes did not vary significantly with trophic ecology. The rate of perfect trials, defined as the proportion of trials for which subjects captured all three prey with a total of three strikes (i.e. one strike per capture), did vary significantly with trophic ecology (*P=*0.0090; Fig. 4A). The three specialized piscivores had the highest rates of perfect trials (*T. salvini* 0.48, *K. umbriferus* 0.37, *P. managuensis* 0.34), and the two lowest rates were from specialized scrapers (*N. nematopus* 0.03, *T. tuba* 0.05).

The first two principal components of the PCA using all nine suction performance variables (total strikes, strikes to each capture, perfect capture rate for each capture, overall perfect single capture rate and perfect trial rate) captured 60.4% and 12.4% of the total variation, respectively (loadings, Table 2; Fig. S1A,B). Higher values along PC1 were associated with fewer total strikes to capture all three zebrafish and higher rates of capture on a first strike attempt. Thus, we interpret the PC1 axis to capture the primary axis of variation in suction-feeding proficiency, with ‘better’ performance corresponding to higher rates of single-strike capture success and fewer strike attempts required to successfully capture an evasive prey. Higher values along PC2 were most strongly correlated with high perfect capture rates on the third fry captured, higher average strikes to capture the second fry and lower average strikes to capture the third fry. PC1 was significantly related to ecological reliance on evasive prey (PGLS: *P*=0.0489); higher reliance on evasive prey was associated with higher values along PC1. The species with highest PC1 score was the specialized piscivore *T. salvini*, and the lowest was the specialized scraper *N. nematopus*.

### Benthic-feeding performance

The amount of food consumed by each species as a proportion of its total body mass ranged from 0.07% (*P. managuensis –* specialized piscivore) to 0.4% (*N. nematopus* – specialized scraper). The mean number of bites taken per trial varied substantially, from 17 (*P. managuensis*) to 105 (*N. nematopus*), and showed a strong inverse relationship with ecological reliance on evasive prey (PGLS: *P*=0.0096; Fig. 4B). The smallest bites were taken by *T. tuba*, a specialized scraper (0.0017% total body mass), and the largest were taken by *T. salvini*, a specialized piscivore (0.011%). We observed a significant, negative relationship between bite size and biting rate (PGLS: *P*=0.0015), and a significant, positive relationship between biting rate and mass consumption (PGLS: *P=*0.0212).

The primary axis of variation in the PCA performed using the three benthic performance variables (PC1=58.9%; Fig. 4C) was most closely related to the bite count (higher PC1 values correspond to higher bite counts), but was influenced by all three variables: bite count, mass consumed (relative to body mass) and bite size (relative to body mass) (loadings, Table 3; Fig. S1C,D). We interpret this PC axis to represent a gradient of increasing benthic-feeding performance, where higher performance is associated with higher rates of biting, larger bites and greater overall mass consumption per unit time. The second principal component (PC2=39.2%) was related to a combination of bite size and total mass consumed (relative to body mass), with higher values corresponding to larger bites and higher total mass consumption. Position along PC1 was related to trophic ecology, with higher values associated with lower reliance on evasive prey. The three specialized piscivores exhibited the lowest scores along the first principal component (*T. salvini*, *K. umbriferus*, *P. managuensis*), and three of the four highest positions were occupied by two algae scrapers and a strict herbivore (*N. nematopus*, *T. tuba*, *H. multispinosa*). PGLS regression of evasive prey reliance against the first principal component revealed a significant positive relationship (*P=*0.0054).

**Table 3.** Loadings for the first and second principal components of the benthic-feeding performance PCA

Variables	PC1 loadings	PC2 loadings
Mass consumed	0.498	0.679
Bite size	−0.442	0.734
Bite count	0.746	−0.017

PC1=58.9%, PC2=39.2%.

### Feeding performance tradeoff

**Fig. 5.**
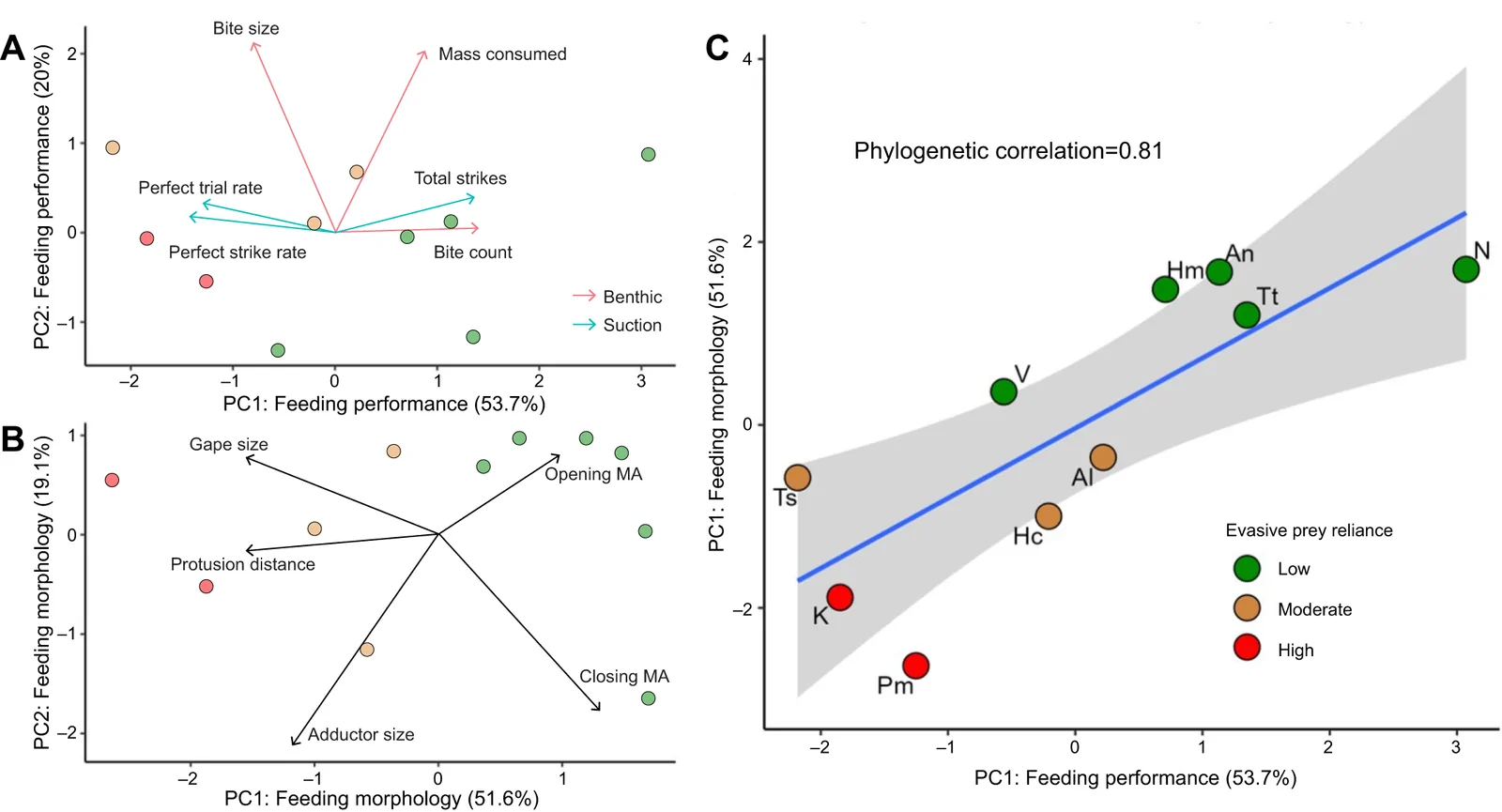
**Feeding performance and trophic morphology in 10 heroine cichlid species.** (A) Overall feeding performance PC1 versus PC2 from PCA, with loadings. (B) Feeding morphology PC1 versus PC2, with loadings. (C) Significant relationship between feeding performance PC1 and feeding morphology PC1. For species abbreviations, see Fig. 4.

We consider the relationship between the opposing axes of feeding performance in two ways. First, we report a significant relationship between PC1 of suction-feeding performance and PC1 of benthic-feeding performance (PGLS: *P*=0.0003; Fig. 4C). Proficient suction-feeding performance (e.g. with PC1 scores associated with higher perfect trial rates and lower total strikes) was associated with poorer benthic-feeding performance (e.g. with PC1 scores associated with low bite counts and low mass consumption). We also performed a principal components analysis using six feeding performance variables, combining three benthic-feeding performance variables (bite size, mass consumed, and bite count) and three suction-feeding performance variables (total strikes, perfect strike rate, and perfect trial rate). Along the primary axis of variation from this analysis (PC1=53.7%; Fig. 5A), we observe a similar inverse relationship between benthic-feeding performance (bite count) and all three measures of suction-feeding proficiency (low total strikes, high perfect trial rate, high perfect strike rate). The second principal component (PC2=20%) mostly described variation in bite size and mass consumption during benthic-feeding trials and separates species with large bites and high mass consumption from those with small bites and lower mass consumption.

### Oral jaw morphology

There was a significant, positive relationship between relative ecological reliance on evasive prey and relative gape radius and relative jaw protrusion, respectively (PGLS: *P=*0.0061, *P<*0.0001). We found a significant, positive relationship between evasive prey reliance and relative adductor muscle mass (*P=*0.0345). There was a negative relationship between the mechanical advantage of jaw closing and reliance on evasive prey (*P=*0.0256). We did not detect a relationship between opening mechanical advantage and trophic ecology. There was also a significant, positive relationship between relative jaw adductor mass and the relative length of the closing in-lever arm (*P=*0.0011), but not between adductor mass and the opening in-lever arm, or between adductor mass and either opening or closing mechanical advantage.

The first PC in a PCA of all five morphological variables (PC1=50.1%) was most strongly associated with relative gape, relative protrusion and mechanical advantage of closing (Fig. 5B), and reflects an axis of diversity between species with large oral gapes, high jaw protrusion and lower closing mechanical advantage (low PC1 values), versus those with small oral gapes, relatively low jaw protrusion and higher closing mechanical advantage (high PC1 values). The second PC (PC2=20.2%) was most strongly associated with the mechanical advantage of closing and the relative mass of the adductor muscles. We report a significant relationship between PC1 of oral jaw morphology and reliance on evasive prey (*P*<0.0001).

### Morphology versus feeding performance

PGLS regression revealed a significant relationship between PC1 in the combined feeding performance PCA and PC1 of morphology (*P*=0.0057; Fig. 5C). We also report significant relationships between PC1 of both benthic-feeding performance and suction-feeding performance against PC1 of oral jaw morphology (PGLS: *P*=0.0007, *P*=0.0347). We found significant relationships between closing mechanical advantage and benthic-feeding performance PC1, bite count and relative mass consumed (*P*=0.0147, *P*=0.0188, *P*=0.0049). We did not detect significance in the relationships between the mechanical advantage of opening and PC1 of suction performance, total strikes per trial, perfect trial rate or perfect strike rate. We did, however, detect significance in the positive relationship between adductor muscle mass and rate of perfect suction trials (*P*=0.0285). We further detected significant relationships between oral jaw gape size and two benthic-feeding performance metrics: mass consumed per bite in benthic trials, relative to body mass (positive, *P*=0.0008), and mean biting rate (negative, *P*=0.0015).

## DISCUSSION

We measured feeding performance in 11 heroine cichlid species spanning a range of ecomorphological diversity to test a longstanding tradeoff hypothesis between suction-based evasive-prey capture and biting-based attached-prey capture. We found strong support for the suction–biting tradeoff: species with better suction-feeding performance generally exhibited poorer benthic-feeding performance, and vice versa. Our findings broadly recapitulate expectations from the ecomorphological paradigm, in which morphology, trophic ecology and feeding performance covary in predictable ways. Species with larger oral-jaw gapes and greater jaw protrusion exhibited superior suction-feeding performance, including a higher probability of capturing evasive prey on the first strike, but showed reduced performance feeding on an attached food compared with species with smaller gapes and less protrusible jaws. However, our analyses also revealed substantial diversity among attached-prey specialists, suggesting that benthic feeding encompasses multiple functional profiles. Although we were unable to quantify suction-feeding performance in one species (*C. bocourti*), all 11 species readily consumed both attached and evasive prey over the course of the study, highlighting the trophic versatility that characterizes the Cichlidae.

The suction–biting tradeoff has traditionally been interpreted through the lens of force–motion tradeoffs in the teleost oral jaws. Previous work has attributed this tradeoff to variation in mandibular mechanical advantage, whereby high mechanical advantage enhances force transmission at the expense of motion transmission, whereas low mechanical advantage favors rapid jaw movement at the expense of force transmission (Burress and Muñoz, 2023; Westneat, 1994). Under this framework, evasive prey capture is expected to favor highly kinetic feeding strikes involving rapid movements of the jaws and buccal cavity (Ferry et al., 2015; Lauder, 1985; Martinez et al., 2018; Wainwright et al., 2015), while attached-prey capture is expected to favor greater transmission of forces from the jaw adductors, through the mandible, to the prey (Wainwright and Turingan, 1993). Consistent with these expectations, oral-jaw gape and protrusion distance were both associated with improved suction-feeding performance and reduced biting performance, indicating that these traits are subject to multifunction performance tradeoffs. Our results are also consistent with recent work demonstrating that attached-prey specialists employ fundamentally different suction mechanics to those of piscivorous cichlids. Rather than the anterior-to-posterior wave of cranial expansion characteristic of generalized suction feeding, algae-specialized cichlids exhibit a more synchronous pattern of head expansion that appears to facilitate efficient transport and retention of detached algae while simultaneously reducing performance during evasive prey capture (De Ridder et al., 2025). Together, these findings suggest that the suction–biting tradeoff extends beyond craniofacial morphology to coordinated changes in feeding kinematics, with morphological specialization accompanied by evolutionary shifts in the timing of cranial expansion.

Importantly, not all morphological correlates of feeding performance contributed equally to the suction–biting tradeoff. Whereas gape size and protrusion distance were associated with opposing effects on suction and biting performance, other traits improved performance along one axis without substantially affecting the other. For example, jaw adductor mass was positively associated with suction-feeding performance but unrelated to benthic-feeding performance, while jaw-closing mechanical advantage was positively associated with benthic-feeding performance but not suction-feeding performance. These findings suggest that the suction–biting tradeoff involves a set of functional traits that are themselves subject to multifunction tradeoffs, whereas other aspects of morphology may enhance performance in one ecological context without necessarily imposing costs in another. This interpretation aligns with previous work showing that some lineages can partially circumvent tradeoffs such as the suction–biting tradeoff through morphological features that decouple improvements in one performance trait from reductions in another (Burress et al., 2020; Burress and Muñoz, 2021; Oufiero et al., 2012; Van Wassenbergh et al., 2007).

Although previous studies have emphasized high bite force as the primary functional endpoint of biting-specialized morphologies (Alfaro et al., 2001; Burns et al., 2024; Westneat, 1994), our results support a more nuanced view of benthic-feeding performance. Attached prey vary widely in their functional properties, and specialization on different forms of attached prey may favor different strategies. Whereas hard or tough prey and prey firmly attached to the substrate may promote the evolution of powerful jaws capable of generating large bite forces, other attached prey such as periphyton, biofilm and small plants may favor alternative solutions. We found evidence of a secondary performance axis among attached-prey specialists, characterized by an inverse relationship between biting rate and mass consumed per bite.

Species such as *N. nematopus* and *T. tuba*, both described as periphyton scrapers (Říčan et al., 2016), exhibited the highest biting rates in our study but relatively low mass consumption per bite. In contrast, *C. bocourti* and *H. multispinosa*, both of which consume vegetation from higher plants, consumed more attached prey per bite while exhibiting lower biting rates. These contrasting profiles were associated with coordinated variation across several traits. High-rate biters possessed smaller oral-jaw gapes and removed less material per bite, whereas large-bite feeders exhibited larger gapes and greater per-bite consumption. Notably, these feeding profiles evolved independently in multiple lineages (Fig. 2), demonstrating repeated convergence on alternative solutions to attached-prey specialization. Thus, we observed relationships between morphology, ecology and performance among benthic-feeding cichlids that demonstrate diet-driven convergence across multiple traits.

The magnitude of biting rates observed in the scraper-like species was striking. On average, our algae-scraping species achieved approximately 34 bites per minute, comparable to peak biting rates reported for grazing marine parrotfishes (33 bites per minute; Choat and Clements, 1993). Previous work has shown that grazing fishes may take thousands of bites over prolonged feeding periods, with one study recording as many as 12,000 bites taken over grazing periods lasting upwards of 12 h per day (Ferreira et al., 1998). This suggests that the ability to sustain high rates of biting can be an important component of ecological success for benthic-feeding taxa. In contrast, we observed the lowest rates of biting among the specialized piscivores, which took an average of 6.2 bites per minute, or approximately one bite every 10 s.

These findings motivate a reconsideration of the role of mandibular mechanical advantage in benthic-feeding performance. High mechanical advantage is traditionally viewed as advantageous because it increases output force. However, the same mechanical properties may reduce the input force required to generate a given output force. For attached-prey specialists feeding on resources that do not require high bite forces, high mechanical advantage may therefore improve feeding performance not by maximizing force production but by reducing the muscular input force necessary to generate a given output force. Under this interpretation, high mechanical advantage could facilitate both high-force and high-rate feeding strategies, albeit for different reasons. Powerful biters may benefit from enhanced force transmission, whereas high-rate biters may benefit from reduced input force costs per bite and the ability to sustain repeated feeding events over long periods. Although direct measurements of feeding energetics are needed to evaluate this hypothesis, it offers a potential explanation for why our benthic-feeding species exhibited a relatively high closing mechanical advantage despite lacking the enlarged jaw adductors typically associated with forceful biting.

We propose that bite rates are constrained by two primary factors: strike duration, defined as the time from the beginning to the end of a feeding strike, and strike latency, the interval between successive strikes. Whereas strike duration is likely a function of jaw mechanics, where mobility-optimized jaws probably execute faster feeding strikes than force-modified jaws, strike latency is more likely to be a function of behavior. We suspect that among benthic feeders, biting rates are constrained by a combination of jaw morphology and bite kinesis, where higher biting rates are expected to correlate with smaller oral jaws, less strike kinesis and shorter feeding strikes. This interpretation is supported by recent volumetric analyses of cichlid feeding mechanics, which found that algae-feeding species generate repeated, relatively low-amplitude, synchronous cranial expansions rather than the traveling expansion wave typical of suction-feeding fishes (De Ridder et al., 2025). Such a feeding mode may be particularly well suited for sustaining rapid feeding cycles while minimizing the loss of detached food particles through the gill openings, providing a plausible kinematic mechanism underlying the high bite rates observed in our scraper species.

In contrast, evasive prey capture specialists are unlikely to be constrained by strike duration. Evasive prey capture specialization favors rapid, explosive feeding strikes that allow the jaws to outpace the reaction time and escape speed of evasive prey. Instead, we suspect that biting rates among non-benthic feeders are constrained by strike latency, or the time between feeding strikes. This is unlikely to be a function of jaw mechanics, and may instead reflect differences in strike selectivity between evasive and attached prey specialists. Whereas the probability of capture success from one moment to another is likely to be highly consistent for attached, sessile prey, it likely varies substantially for evasive prey, arising from variation in factors such as prey behavior and position relative to the predator through time. This may present a selective pressure for strike selectivity among evasive prey capture specialists that would not exist among attached prey specialists.

This idea is supported by our observation that evasive-prey specialists differed from other species not in the average number of strikes required to capture prey but in the probability that a first strike resulted in a successful capture. This pattern is noteworthy because prey often alter their behavior following an attempted predation event, potentially reducing predator success on subsequent attacks (Lee et al., 2006; Lett et al., 2014; Mohapatra and Sinha Mahapatra, 2025; Sih et al., 2010). Under natural conditions, unsuccessful strikes may permit prey to seek refuge or flee entirely, increasing the importance of initial strike success. Experimental prey in our trials were comparatively limited in their post-attack options, potentially increasing the effectiveness of repeated attacks, thereby reducing differences among species in the total number of strikes required for capture. This idea is supported by previous work, which found that cichlids hunting evasive prey in enclosures of different sizes successfully captured larger prey in smaller experimental spaces (Christensen, 1996). Thus, behavior appears to play an important role in linking morphology to performance.

The positive association between adductor mass and suction-feeding performance may provide further insight into this pattern. Although large adductor muscles are often associated with forceful biting, they may also facilitate rapid closure of the larger oral jaws found in evasive prey specialists, and have been observed in association with specialized piscivory and enhanced suction performance in other ecomorphological studies (Burns et al., 2024; Carroll and Wainwright, 2009). While much attention has focused on the role of jaw opening and protrusion in suction feeding, successful capture also requires rapid retention of prey once it has entered the oral cavity. Larger jaw adductors may therefore contribute to explosive feeding strikes and improve first-strike success. However, highly kinetic strikes involving the rapid displacement of relatively large jaw elements may also be energetically costly, potentially helping to explain why suction-specialized species exhibited relatively poor performance in high-rate benthic-feeding contexts.

A second driver of variation in jaw adductor morphology may be the relative length of the closing in-lever. We found a positive relationship between jaw adductor size and closing in-lever length. For a given angular displacement of the mandible, a longer in-lever requires greater muscle extension, resulting in a greater likelihood of the muscle functioning at less than peak force capacity because of its length (Polet and Labonte, 2024). Viewed in this context, variation in in-lever length results in a trade-off between force and displacement because of both its effect on mechanical advantage of the lever and its interaction with the adductor muscle's force–length relationship.

In summary, we provide experimental evidence for the suction–biting tradeoff in a cichlid adaptive radiation, reinforcing the importance of multifunction tradeoffs in shaping trophic diversification. Our findings suggest that this tradeoff arises primarily from morphological traits that simultaneously influence suction and biting performance, whereas other traits contribute to performance variation without necessarily imposing tradeoffs. We also demonstrate that attached-prey specialization itself encompasses multiple performance profiles, including both high-rate and large-bite feeding archetypes that appear to have evolved repeatedly among heroine cichlids. Finally, we propose that the relationship between morphology and feeding performance may depend not only on force production and motion transmission but also on the energetic and behavioral demands of different feeding strategies. Future work integrating feeding energetics, kinematics and performance will help clarify how these factors interact to shape trophic diversification across fishes. Recent evidence that algae-specialized cichlids employ an alternative pattern of cranial expansion during feeding suggests that evolutionary diversification of feeding systems involves coordinated changes in morphology, kinematics and performance, rather than modifications to any single component alone (De Ridder et al., 2025).

## Supplementary Material

10.1242/jexbio.252347_sup1 Supplementary information
