# Supplementary material for: Experimental evidence for a suction–biting performance tradeoff in Mesoamerican cichlids
Source: J Exp Biol. 2026 Sep 9;229(17):jeb252347. doi: 10.1242/jeb.252347 (PMC13615685; doi:10.1242/jeb.252347)
Supplement: Supplementary file 1 [file jexbio-229-252347-s1.pdf]

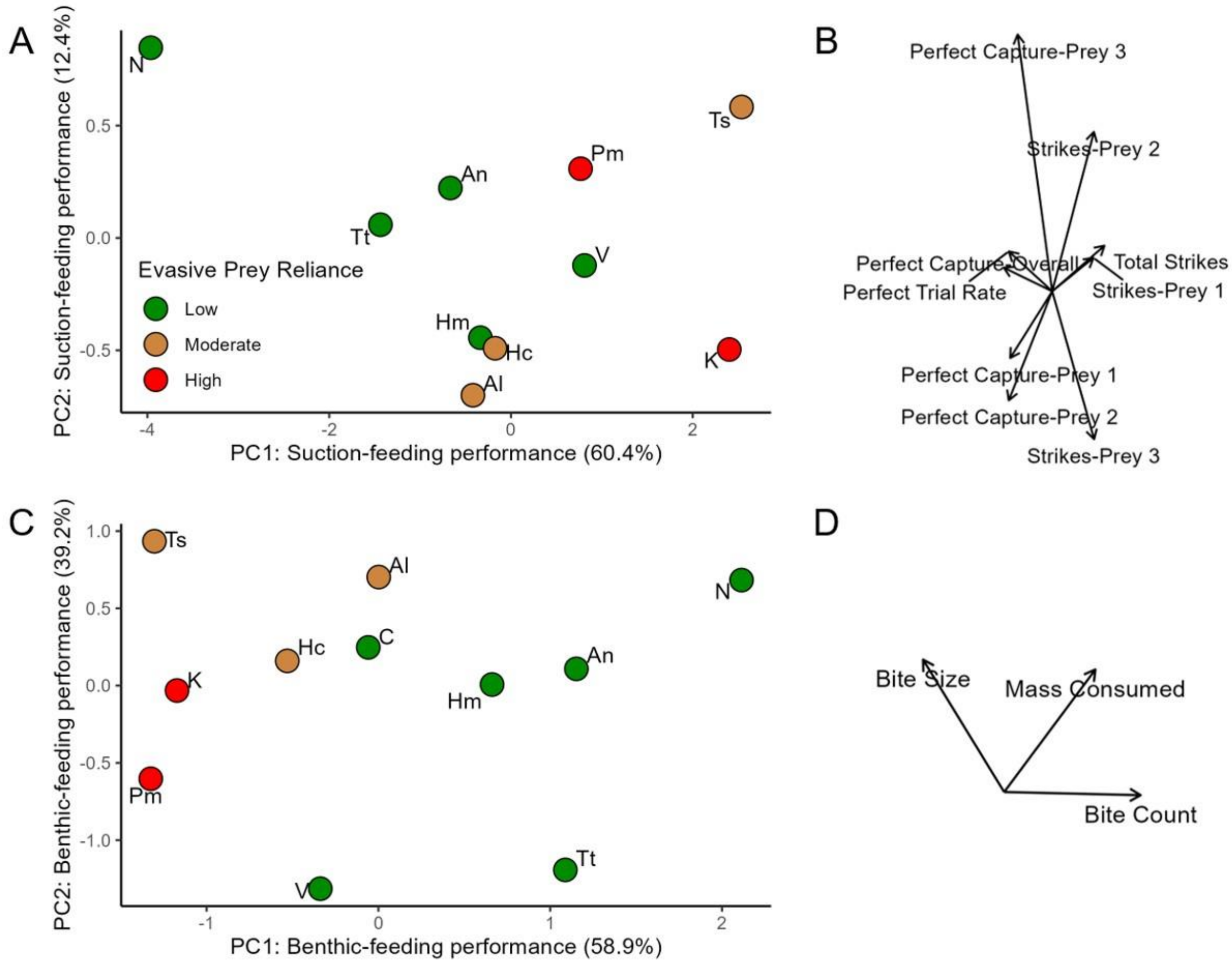

Fig. S1. (a) Suction-feeding performance PCA. (b) Suction-feeding performance PCA loadings. (c) Benthic-feeding performance PCA. (d) Benthic-feeding performance PCA loadings.

Journal of Experimental Biology • Supplementary information

Table S1. Morphological trait measurement definitions.

Measurement	Definition
Standard length	Distance between caudal peduncle and distal tip of oral jaws, closed & retracted
Body mass	Mass of specimen, euthanized, preserved in ethanol, patted dry
Adductor mass	Mass of adductor muscles, dissected from preserved specimen, patted dry
Oral jaw protrusion	Distance between distal tip of upper jaw and anterior margin of the eye, difference between when jaws are fully retracted and fully protruded
Oral jaw vertical gape	Distance between distal tips of the upper and lower jaws when fully protruded
Oral jaw horizontal gape	Distance between the corners of the oral jaws when fully protruded
Opening in-lever	Distance between center of articular-quadrate joint and tip of ascending process of angular-articular
Closing in-lever	Distance between center of articular-quadrate joint and distal tip of retroarticular
Out-lever	Distance between center of articular-quadrate joint and tip of distalmost tooth of the lower jaw

Journal of Experimental Biology • Supplementary information
